# P105: Efficacy of a virucidal surgical handrub

**DOI:** 10.1186/2047-2994-2-S1-P105

**Published:** 2013-06-20

**Authors:** Y Kumashita, P Mohan, M Mine, M Yamamoto

**Affiliations:** 1 Biochemical laboratory, Saraya CO. LTD., Osaka, Japan

## Introduction

Various alcohol (EtOH (ethanol), IPA (isopropanol) and PA (n-propanol)) and acid (Phosphoric acid and Lactic acid) combinations were evaluated to come up with a hand antiseptic formula that is virucidal (EN14476) and effective as a surgical handrub (EN12791) at a short contact time.

## Methods

Screening tests using bacteriophage MS2 was performed, and combinations with good results were identified. These combinations were tested in accordance with EN14476 againt poliovirus, adenovirus, and feline calicivirus. The alcohol and acid combination with the best results was chosen, and an emollient was then added. This formulation was evaluated in accordance with several EN standards including EN12791. Skin irritation was also investigated.

## Results

The mixture of EtOH (ethanol), PA (n-propanol) and Phosphoric acid exhibited the greatest efficacy among the combinations tested. When tested against EN standards for hand antiseptic, it was shown to meet EN14476 virucidal handrub and EN12791 surgical handrub requirements in relatively short contact times, less than 1 minute and 1 minute, respectively.

## Conclusion

The handrub formulation containing EtOH, PA and Phosphoric acid exhibited excellent bactericidal and virucidal efficacy at a relatively short contact time and had lower skin irritation, suggesting that it is suitable for fast-acting hand disinfection.

## Disclosure of interest

None declared

